# Chinese medicine targets cellular autophagy against cardiovascular diseases: research progress and future prospects

**DOI:** 10.3389/fcvm.2025.1585407

**Published:** 2025-05-26

**Authors:** Zhengyu Chen, Jinjin Dou, Xiwu Zhang

**Affiliations:** ^1^ Graduate School, Heilongjiang University of Traditional Chinese Medicine, Harbin, China; ^2^ Department of Cardiovascular, The Fourth Hospital of Heilongjiang University of Traditional Chinese Medicine, Harbin, China; ^3^ Experimental Training Centre, Heilongjiang University of Traditional Chinese Medicine, Harbin, China

**Keywords:** cardiovascular diseases, autophagy, Chinese herbal extract, signaling pathway, Chinese medicine compound

## Abstract

Cardiovascular diseases (CVDs) pose a serious threat to human health and represent one of the leading causes of death worldwide. Cellular autophagy, an essential intracellular self-degradation and homeostasis maintenance mechanism, plays a pivotal role in the pathogenesis of cardiovascular diseases. Traditional Chinese Medicine (TCM), with its unique theoretical framework and therapeutic principles, has demonstrated remarkable efficacy in CVDs management, garnering increasing scientific attention. In recent years, growing research attention has focused on TCM's autophagy regulation for CVDs treatment. However, most studies remain limited to cellular and animal models, with insufficient clinical data and unclear specific metabolic pathways and targets. Therefore, it is imperative to (1) investigate autophagy mechanisms in depth (2), explore methods for autophagy balance, and (3) clarify drug interactions to establish a foundation for clinical applications. This article comprehensively summarizes relevant research findings, provides an in-depth discussion of TCM's mechanisms in autophagy regulation for CVDs treatment, reviews current research status, and outlines future development trends, aiming to offer valuable theoretical foundations and therapeutic strategies for clinical CVDs management.

## 1 Introduction

According to epidemiology, CVDs refer to the main causes of global mortality. Despite significant advancements that have occurred in the treatment and prognosis of these diseases, the annual cost spent on therapy is still very high (1). Latest statistics from the American Heart Association reveal that 49.2% of adults aged 20 and above in the United States suffered from CVDs (2). Such diseases mainly include atherosclerosis (AS), hypertension, heart failure (HF), myocardial infarction (MI), arrhythmia, and dilated cardiomyopathy (DCM) (3, 4). Autophagy is signified by the generation of autophagic vesicles with a double-layer membrane structure to wrap part of the cytoplasm and intracellular components and fuse with lysosomes to constitute an autophagic lysosome for degradation of its wrapped contents, aiming to fulfill the intracellular metabolic demands and the renewal of specific organelles. Autophagy represents a common cell death mode different from apoptosis, pyroptosis, and ferroptosis (5), and it is also involved in disease defense and stress regulation processes. Autophagy is involved in the emergence and development of different CVDs, affecting cardiac function and the prognosis of diseases. Autophagy at a physiological level acts as a defense for cardiomyocytes against environmental stimuli, while excessive autophagy may cause CVDs (6). Studies confirmed that abnormal autophagy contributed to abnormal metabolism of cardiomyocytes (7), myocardial dysfunction, and induced cardiovascular diseases (8). TCM boasts a history spanning over 2,000 years and has been widely applied in clinical practice (9). Rooted in the holistic concept of TCM theory, it achieves systemic regulation of the cardiovascular system. Chinese herbal formulas contain multiple active components that exert therapeutic effects through multi-target synergistic modulation. For patients requiring long-term cardiovascular treatment, TCM demonstrates distinct advantages: its treatment costs are substantially lower than comparable Western medicines, while its relatively low toxicity results in significantly fewer adverse drug reactions than conventional pharmaceuticals (10). Research have indicated that autophagy can be regulated by TCM in a bidirectional manner, including both improving the resistant ability of cardiomyocytes to non-zero stimuli and lessening the emergence of adverse cardiovascular events and excessive autophagy to enhance the survival rate of cardiomyocytes (11, 12). This paper mainly reviews the relevant mechanisms of autophagy in AS, DCM, HF, MI and reperfusion, and AS and research progress of TCM in treating CVDs by regulating autophagy, aiming to provide more favorable references for the clinical therapeutics of CVDs.

## 2 Autophagy and its related mechanisms

### 2.1 What is autophagy

The term autophagy first appeared in 1963, proposed by Nobel Prize winner Christian de Duve. Autophagy refers to an effective homeostasis pathway by promoting the degradation and reclamation of cellular substances. Cellular autophagy includes non-selective and autophagy (13). At present, we know three kinds of non-selective autophagy, containing microautophagy, macroautophagy, and molecular chaperone-mediated autophagy, among which macroautophagy is the most specifically investigated and the most intensively applied. Macroautophagy involves the encapsulation of cytoplasmic material within double-membraned autophagosomes. These specialized vesicles subsequently combine with lysosomes, enabling enzymatic degradation of their contents into basic molecular components (14). Microautophagy: the membrane of the lysosome invaginates inward to directly enclose the cellular contents and degrade them within the lysosome (including KFERQ-labeled proteins or large amounts of cytoplasmic contents). Chaperone-mediated autophagy(CMA): cytoplasmic proteins bind to chaperones and are then transported into the lysosomal lumen and digested by lysosomal enzymes. Unlike the above autophagy, chaperone-mediated autophagy functions without vesicles. This type of autophagy is characterized by high selectivity and usually relies on the chaperone protein Hsc70 to specifically decompose proteins due to its recognition pentapeptide motif (KFERQ-like). LAMP2A recognizes the bare KFERQ group of the binding protein and assists in guiding it into the lysosome for degradation, serving as a receptor protein on the lysosomal membrane (15). However, many studies now also show that specific autophagy can eliminate some harmful cytoplasmic objects, including dysfunctional organelles (lipophagy, mitophagy, reticulophagy, lysophagy, and nucleophagy), invading pathogens (xenophagy), and protein aggregates (aggrephagy) (16, 17).

### 2.2 Autophagy—related mechanisms

**Figure 1:**
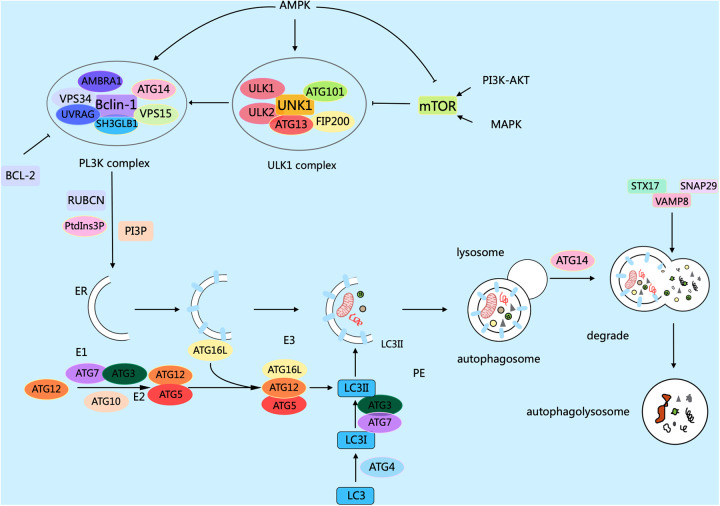
Mechanisms related to cellular autophagy. mTOR, AMPK, and ULK1 are key protein kinases in autophagy and function in the initiation and regulation of autophagy. The PI3K complex contributes to autophagy and other physiological processes, and its main members include Beclin-1, VPS34, ATG14, VPS15, and other proteins. The different members collaborate with each other to fulfill their biological functions. Arrows indicate stimulation; T-arrows indicate inhibition. Created with MedPeer (medpeer.cn).

Autophagy in eukaryotic cells can be divided into five stages: (1) the initiation stage; (2) the nucleation stage (formation of the phagophore); (3) the formation stage (the phagophore expands itself to form a double-membrane vesicle); (4) the stage of autophagosome-lysosome fusion; (5) the lysosomal degradation stage (lysosomal proteases degrade the enclosed substrates) (18, 19). The modulation of autophagy involves numerous factors, molecular pathways, and autophagy-related genes (*ATG*) such as *ATG1*, *ATG3*, *ATG5*, *ATG6/Beclin1*, *ATG7*, *ATG8/LC3*, *ATG12*, etc. contribute significantly to autophagy (20). Research has confirmed that over 35 *ATGs* were discovered in both yeast and mammals (21). The formation of autophagosomes begins with the generation of phagophores. The activation of the ULK1/Atg1 complex initiates autophagy. This process is regulated by the mammalian target of rapamycin complex 1 (mTORC1) and AMP-activated protein kinase (AMPK), which serve as the primary nutrient and energy sensors in cells. Meanwhile, the Unc-51 complex macromolecule that initiates the phagophore consists of ATG13, Ulk1, or Ulk2 (the mammalian orthologs of ATG1, the 200 kD focal adhesion kinase family-interacting protein (FIP200), and ATG101 (22). Activated ULK1 can activate the marker molecule yeast ATG6 homolog (Beclin-1) in the autophagy initiation stage, enabling it to bind to ATG14, VPS15, and VPS34 proteins to form the class III phosphatidylinositol 3-kinase (PI3K) complex, which promotes the autophagy proteins targeting to autophagic vesicles and stimulates the production of the cup-shaped isolation membrane. However, B-cell lymphoma-2 (Bcl-2) can bind to Beclin-1 to inhibit the occurrence of autophagy (23). ULk1 phosphorylates Beclin-1 at serine 14, thus activating the VPS34 complex. Research has confirmed that there are various regulatory interactors of the VPS34 complex, including UVRAG, that promote the catalytic activity of VPS34, SH3GLB1, and AMBRA1, as well as RUBCN and Bcl-2 that inhibit the catalytic activity of VPS34. This complex, through the formation of phosphatidylinositol 3-phosphate (PI3P), and subsequent engagement with the PI3P-binding proteins of the WIPI family, contributes to the expansion of the phagophore (24). PI3P aids in the expansion of the phagophore by promoting the translocation of autophagy proteins to the phagophore assembly site, such as ATG18, ATG20, ATG21, and ATG24. During this process, the phagophore sequesters cytoplasmic cargo, matures, and closes. ATG8 family members, including MAP1LC3, are known as LC3. After cleaving LC3 into LC3I by the ATG4 enzyme, LC3I is further converted into LC3II with the involvement of the ubiquitin-like enzymes ATG3 and ATG7. Meanwhile, AGT12 binds to AGT5, which is mediated by the E1-like protein Atg7 and E2-like proteins ATG10 and ATG3. Then, the complex of AGT12 and ATG5 assists in the elongation of the mature phagophore by interacting with ATG16l. ATG16l acts as an E3-like enzyme and promotes the lipidation of LC3 by conjugating with phosphatidy-lethanolamine. Finally, after LC3II binds to phosphatidylethanolamine (PE) to form a complex, it can act on the ATG12-ATG5-ATG16L1 complex and participate in the extension of the autophagosome membrane, ultimately forming an autophagosome with a double-membrane structure. Finally, the mature autophagosome combines with the lysosome, permitting lysosomal enzymes to degrade and recycle the cargo materials. After maturation, the autophagosome merges with the lysosome, mediated by the ATG8 family proteins and the autophagy-related SNARE proteins STX17, SNAP29, and VAMP8 via an ATG14-dependent mechanism (25). LC3II interacts with P62 to transport autophagic contents into the autolysosome, where they are further degraded into small molecules by hydrolases for reuse by the body's cells (26) (Figure 1).

### 2.3 Autophagy-related pathways

The biological activity of autophagy is correlated to mTOR-dependent and non-dependent signaling regulation pathways (27). The mTOR non-dependent pathways: AMPK, PI3K, Ras-MAPK, p53, PTEN, endoplasmic reticulum stress. mTOR-dependent pathways: (1) PI3K/Akt/mTOR pathway functions in cell proliferation, apoptosis, oxidation, and angiogenesis. Phosphatidylinositol-dependent kinase 1 mediates the phosphorylation-dependent activation of Akt for autophagy, while hyperactivation of phosphatidylinositol-PTEN leads to autophagy by the inhibition of this pathway (28). The PI3K/Akt/mTOR axis was identified to be highly activated in CVDs, and a decrease in mTOR expression can play a protective role against injured cardiomyocytes (29). (2) AMPK, a highly conserved serine/threonine protein kinase, is a vital kinase involved in detecting cellular energy levels and managing cell signaling during autophagy. The AMPK/mTOR signaling pathway contributes to the process of cell growth and proliferation, energy metabolism, and autophagy regulation (30). In addition, the phosphorylation of TSC2 and Raptor by AMPK, both mTOR-associated regulatory proteins, results in the inactivation of mTORC1 and triggers autophagy by phosphorylating the serine 93 and 96 sites of the autophagy gene Beclin1, which affects the activity of VPS34 (31). Some research has confirmed that the AMPK/mTOR signaling pathway can be involved in the advancement of CVDs, containing myocardial ischemia/reperfusion injury(MIRI), AS, myocardial hypertrophy, etc., Studies the regulation of autophagy (32, 33). (3) MAPK-related pathways can be affected by diverse extracellular stimuli, leading to different biological responses based on their mutual regulation. JNK, ESCRK, and MAPK-associated kinase are the main components of MAPK-associated signaling pathways. Recently, increasing studies revealed that the MAPK family contributes to the regulation of autophagy (34). Some studies even confirmed that the MAPK/mTOR axis can participate in the atherosclerotic process by regulating autophagy (35). Other pathways: (1) Hippo-related pathway: increasing findings suggest that the Hippo pathway is closely related to autophagy, yet regulates the survival and mortality of cardiomyocytes (36). The upstream component MST1 of this pathway can inhibit the cytoplasmic expression of Beclin-1 and/or Silence Information Regulator 1 (SIR) through direct phosphorylation of Beclin-1 and/or inhibition of MST1 (37). (2) Wnt/β-catenin-related pathway: As a glycoprotein, Wnt can function through autocrine or paracrine secretion, and communicate with specific cell surface receptors, causing the aggregation of β-catenin, which then regulates related gene expression and intervenes in the process of cardiovascular disease. Studies have confirmed that dysregulation of Wnt/β-catenin pathway activity may be linked to cardiac hypertrophy and the process of cardiomyocyte remodeling in the pathological state (38), and may also participate in vascular calcification, a cardiovascular disease pathology, through the regulation of autophagy (39).

## 3 Autophagy and cardiovascular disease

### 3.1 Autophagy and atherosclerosis

**Figure 2:**
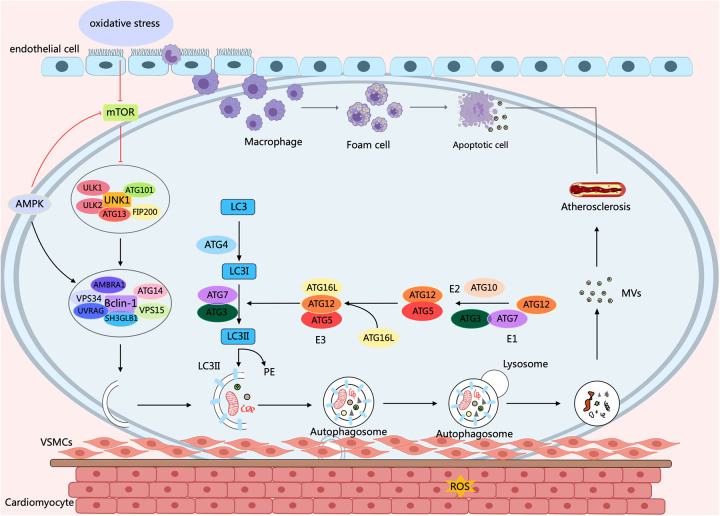
Autophagy process and its effect on AS. Under oxidative stress conditions, a variety of cells, concerning endothelial cells, macrophages, and smooth muscle cells, ultimately contribute to the molecular mechanisms of AS through diverse signaling pathways of mTOR and AMPK, as well as cellular processes of autophagy and apoptosis. Arrows indicate stimulation; T-arrows indicate inhibition. Created with MedPeer (medpeer.cn).

Atherosclerosis (AS) is a chronic inflammatory and metabolic disorder affecting the vascular system, which can lead to heart attacks, ischemic strokes, and peripheral arterial diseases. *in vivo* studies demonstrate that rapamycin, an autophagy activator, prevents the organization and development of macrophage-derived foam cell by decreasing lipid content within cells. It helps inhibit AS by reducing macrophage apoptosis through the clearance of dysfunctional mitochondria (40). Increasing evidence indicates that a close correlation exists between dysfunctional autophagy and the overproduction of Reactive Oxygen Species (ROS) in the initiation and advancement of atherosclerotic disease (41). When autophagy is abnormal, apoptosis and necrosis increase, further promoting plaque instability (42). Research has found that the expressions of autophagy markers ATG13 and LC3 in the endothelial cells of the aortic intima with severe a AS are significantly higher than those in the aortic intima endothelial cells without AS (43). Defects in EC autophagy increase VCAM-1, ICAM-1, vascular hemophilic factor, and P-selectin levels, thereby improving macrophage and foam cell infiltration and promoting arterial thrombosis risk (44). However, in advanced atherosclerosis, sustained ox-LDL and inflammatory cytokine exposure trigger excessive autophagy in endothelial cells, resulting in cell death. This promotes plaque instability and rupture, thereby elevating the risk of thrombosis and acute coronary syndromes (45) (Figure 2).

### 3.2 Autophagy and diabetic cardiomyopathy

**Figure 3:**
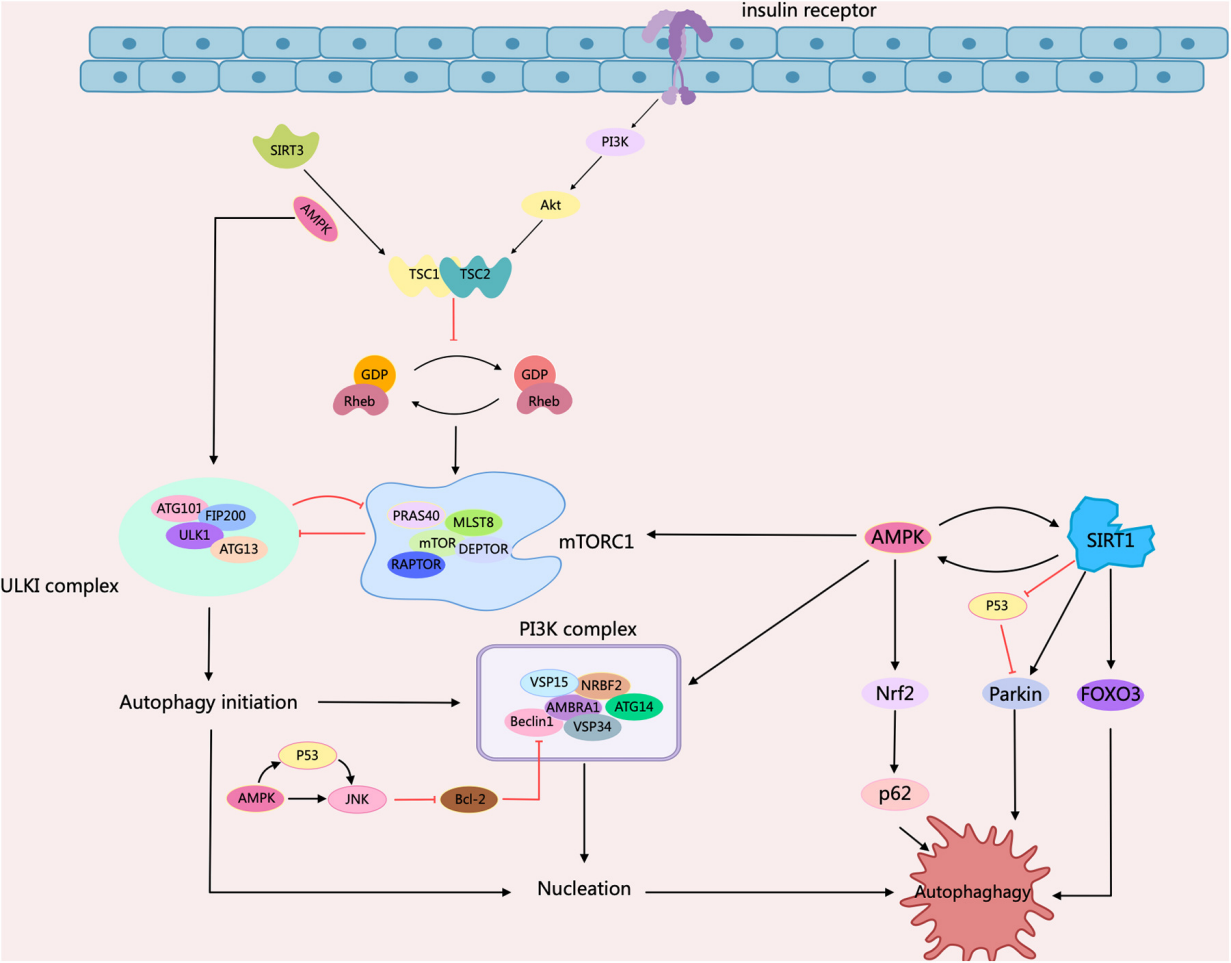
Schematic representation of intracellular signaling mechanisms in diabetic cardiomyopathy regulated by cellular autophagy. When insulin binds to the insulin receptor, phosphorylation of PI3K/Akt recruits Rheb to activate mTORC1. mTORC1 is autophagy-induced by impaired ULK1 activity through phosphorylation of ULK1. It can then promote autophagy by regulating mTORC1 through a negative feedback loop, either through inhibition of mTORC1 or through activation of TSC1/2. AMPK serves as an important regulator, inhibiting the activation of mTORC1 by phosphorylating TSC2 or mammalian ULK1. AMPK supports the activation of JNK1, which restores autophagy by triggering Beclin1-Bcl2 dissociation via Bcl2 phosphorylation. Meanwhile, AMPK interacts with SIRT to phosphorylate FOXO3 and Nrf2 to support autophagy, and mediated p53 deacetylation, inhibiting p53 and inducing autophagy. Arrows indicate stimulation; T-arrows indicate inhibition. Created with MedPeer (medpeer.cn).

Diabetes mellitus (DM) represents a type of chronic disorder involving metabolic dysfunction and featuring hyperglycemia, significantly impacting human health. As the main cardiovascular complications and major causes of death among diabetic patients (46). Diabetic cardiomyopathy (DCM) is an adverse factor for cardiac remodeling, containing myocardial fibrosis, and cardiac hypertrophy, along with early-onset diastolic and late-onset systolic dysfunction. In DCM, the pathophysiological factors concern insulin resistance, mitochondrial damage, metabolic disorders, production of advanced glycation end-products, inflammatory activation, and necrocytosis, etc (47). Numerous studies indicated that signaling pathways such as mTOR/AMPK, FOXOs, Nrf2, SIRTs, Parkin, and *Atg* gene products contribute to the regulation of diabetic cardiomyopathy by cellular autophagy (48, 49). There is evidence showing that the structural and functional disorders of cardiomyocytes in DCM patients are closely related to abnormal cellular autophagy. It has been reported that high-fat feeding inhibits cardiac autophagy in diabetic mice by activating mTOR1, manifested as down-regulated LC3-II expression and up-regulated p62 expression. The inhibition of autophagy in cardiac tissue exacerbates ischemic injury after MI in diabetic mice (50). Another study illustrated the relationship between autophagy and DCM. They found that AMPK activity was inhibited and phosphorylated mTOR expression was increased, leading to the inhibition of cardiac autophagy in T2DM hearts, based on the db/db type 2 diabetic mice model (51). In addition, AMPK contributes to the autophagy of DCM cells. Activated AMPK can induce the dissociation of Bcl-2 from Beclin1, and then regulate autophagy in diabetic myocardial tissue (52, 53). In contrast, impaired insulin signaling in the diabetic milieu can lead to excessive activation of autophagy in the diabetic heart, which may also exert detrimental effects on cardiac function (48) (Figure 3).

### 3.3 Autophagy and myocardial ischemia-reperfusion injury

**Figure 4:**
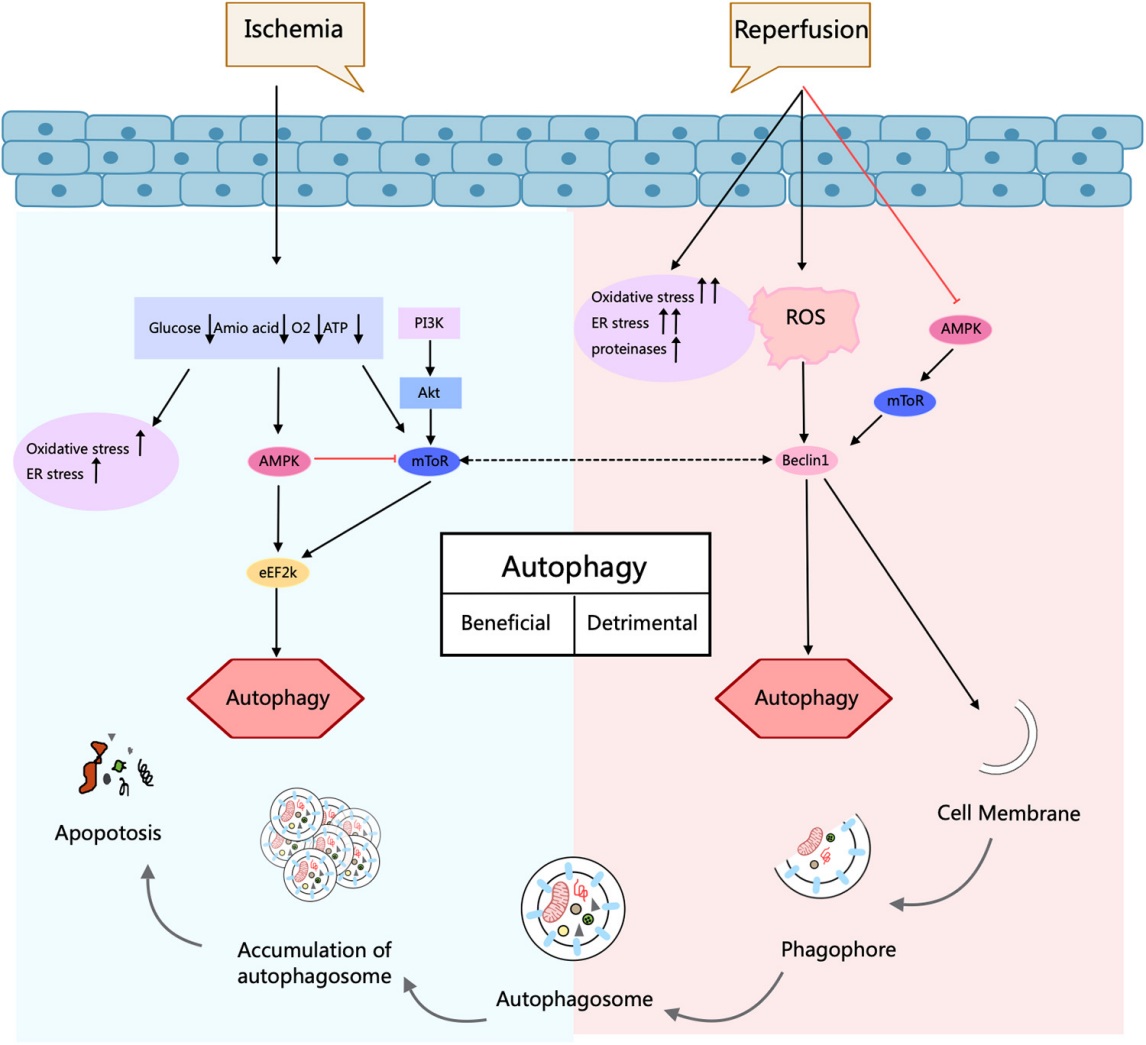
Autophagy process and its role in ischemia/reperfusion injury in cardiomyocytes. Autophagy is a cellular self-degradation process that has a dual role in cardiomyocyte response in ischemia/reperfusion injury. During ischemia, AMPK activation promotes protective autophagy and contributes to cell survival. In contrast, during the reperfusion phase, elevated Beclin1 levels and increased oxidative and endoplasmic reticulum stress drive deleterious autophagy and may exacerbate cellular injury. Thus, depending on the conditions, autophagy can be regulated by different signaling pathways that play different roles in protection or injury. Arrows indicate stimulation; T-arrows indicate inhibition. Created with MedPeer (medpeer.cn).

MI is the main incentive of CVDs-related deaths caused by coronary artery stenosis, spasm, and embolism due to AS. In recent years, both its incidence and mortality rates have risen sharply (54). MIRI is the pathological process of aggravated myocardial injury after the ischemic heart restores blood perfusion. Its pathogenesis is mainly associated with PI3K/Akt, Akt/mTOR, AMPK, and MAPK pathways (55). It has been found that in the MI stage, autophagy can inhibit apoptosis and necrosis, which has a cardioprotective effect; while in the reperfusion stage, autophagy is activated in large quantities, leading to cardiomyocyte death and increased myocardial injury (56). In a study using the right atrial appendage of human hearts required after cardiac arrest, *ATG* expression was assessed by PCR. It showed that Ischemia/Reperfusion (I/R) led to an increase in the expression of 11 out of 84 ATG proteins, a down-regulation of 3, and an elevated LC3-II/LC3-I ratio. These findings reveal that I/R of the human heart can result in the initiation of autophagy (57). Furthermore, in MI and reperfusion, surplus ROS accumulation provokes a sustained autophagic response, leading to more apoptosis and impaired heart function, which may occur through AMPK/mTOR (58). AMPK controls FOXO3-driven autophagy under hypoxic conditions in cardiomyocytes (57). It regulates fuel supply and energy-generating pathways to address the metabolic requirements during MI and reperfusion (Figure 4).

### 3.4 Autophagy and heart failure

**Figure 5:**
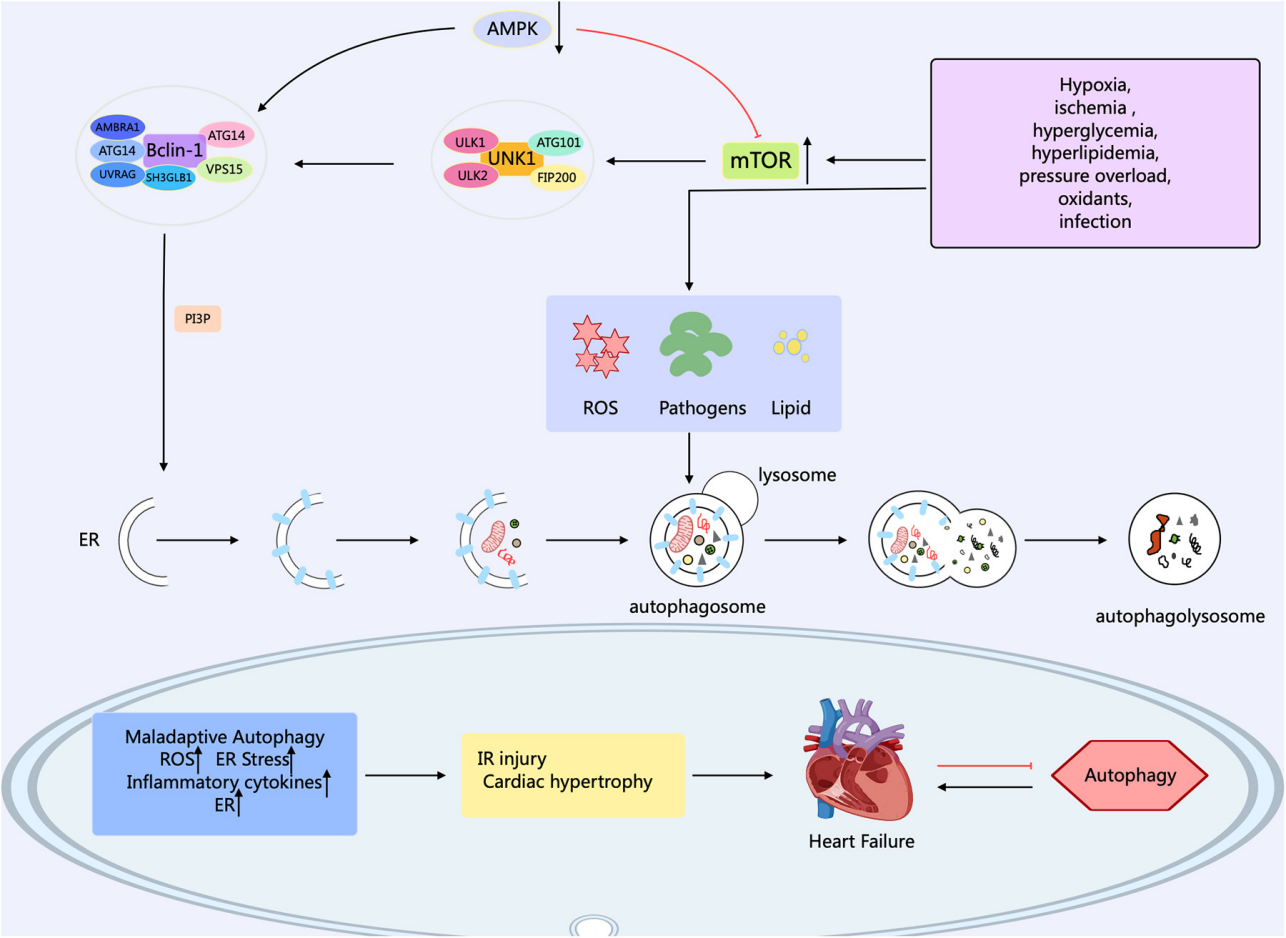
Role of autophagy process in HF. Under the conditions of various disease-influencing factors, signaling pathways of mTOR and AMPK are activated or inhibited, and the mechanisms of autophagy are regulated in a variety of ways to promote or inhibit the advancement of HF. Arrows indicate stimulation; T-arrows indicate inhibition. Created with MedPeer (medpeer.cn).

Chronic HF is induced by decreased cardiac output due to neurohumoral imbalance and cardiac remodeling, which may be associated with inflammation, oxidative stress, apoptosis, mitochondrial function, endoplasmic reticulum stress, autophagy, and angiogenesis (59). mTOR suppresses autophagy, whereas AMPK and glycogen synthase kinase-3β (GSK-3β) activate this cellular degradation process in cardiomyocytes. These regulatory mechanisms during endothelial-mesenchymal transition contribute to myocardial fibrosis formation. GSK-3β serves as a positive regulator for autophagy in energy metabolism and endothelial mesenchymal transition in cardiomyocytes, leading to cardiac fibrosis and cardiac hypertrophy (60). Abnormal autophagy has been illustrated in the myocardium of patients with CHF caused by valvular disease, dilated cardiomyopathy, and coronary artery disease. It has been found that chikusetsusaponin IVa (CS) stimulates the action of autophagy through the AMPK/mTOR/ULK1 axis in an isoprenaline-induced myocardial fibrosis model, which significantly attenuates myocardial fibrosis, reduces cardiac index, inhibits infiltrated inflammation, and reduces collagen deposition as well as cardiomyocyte size (61). Another study indicated that increased muscle growth inhibitors in patients with HF inhibited excessive myocardial autophagy and attenuated myocardial hypertrophic features by blocking the AMPK/mTOR axis (62) (Figure 5).

## 4 Traditional Chinese medicine protects against cardiovascular diseases via modulation of autophagy

The pathogenesis of CVDs mainly consists of related mechanisms of metabolic disorders, oxidative stress, inflammatory responses, and cell apoptosis (63). The autophagy process can be initiated when cells encounter the conditions of hypoxia, oxidative stress, inflammation, lipoprotein oxidation, and ROS (31). Autophagy functions in both normal and diseased hearts and helps to maintain normal cardiac function (64). Autophagy plays a role in both normal and diseased hearts, contributing to the maintenance of normal cardiac function (39). It exhibits remarkable bidirectionality-moderate activation or inhibition can regulate disease progression, but its effects are highly dependent on the disease type, stage, and cellular microenvironment. This seemingly paradoxical phenomenon stems from the multifaceted functions of autophagy under different pathophysiological conditions. In addition, activation or inhibition of autophagy may exert diametrically opposite effects on the disease at different pathological stages of the same disease (56, 65, 66).

**Figure 6:**
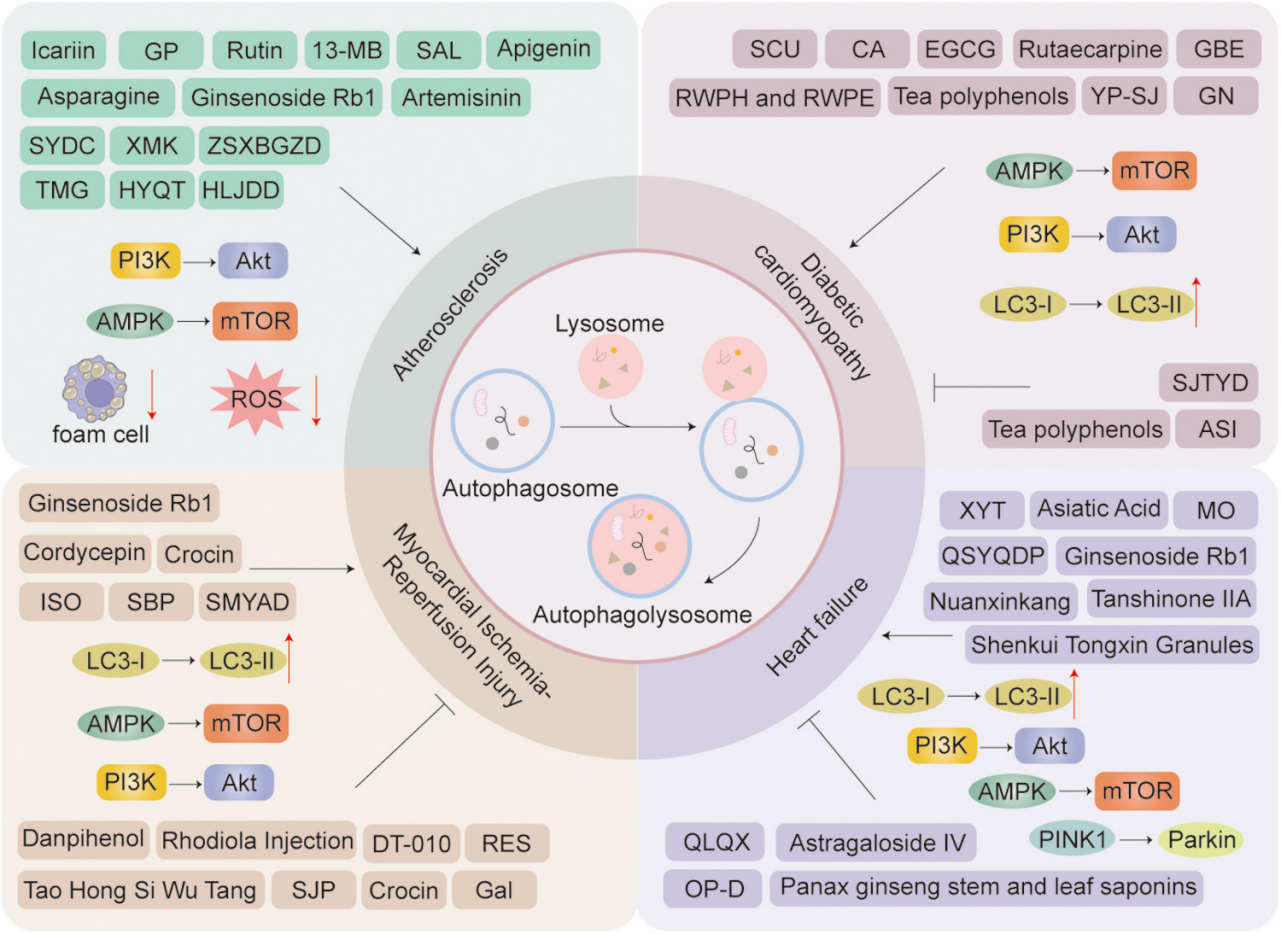
Chinese herbal extract, proprietary Chinese medicines, and Chinese medicine compounds in the treatment of cardiovascular diseases through autophagy: a schematic diagram illustrating several key mechanisms and indicators. Arrows indicate stimulation; T-arrows indicate inhibition.

TCM achieves comprehensive therapeutic effects against diseases through multi-component and multi-target mechanisms, utilizing herbal extracts, proprietary Chinese medicines, and compound formulations. Recent extensive studies have demonstrated thatChinese medicine compounds, Chinese herbal extract, Chinese Patent Medicines, and Chinese Herbal Formulas can effectively and precisely treat cardiovascular diseases by modulating cellular autophagy (Figure 6).

### 4.1 Autophagy activation in the treatment of cardiovascular diseases by Chinese medicine compounds, herbal extracts, patent medicines, and formulations

#### 4.1.1 Atherosclerosis

Wang et al.'s study (67), found that icariin stimulates autophagy by promoting the fusion of autophagosomes and lysosomes, thereby reducing ROS levels. TFEB/Lamin B1 and LC3II/I expression under exposure to ox-LDL, which encourages the combination of autophagosomes and lysosomes, enhancing autophagy, and inhibiting ferroptosis. Meanwhile, *in vivo*, icariin is capable of suppressing pro-inflammatory factors and boosting autophagy. In the experiment, icariin reduced IL-6 and TNF-α levels in the model group. Therefore, icariin is effective in inhibiting ferroptosis, controlling AS, and managing the secretion of pro-inflammatory factors. Immunoprecipitation assays indicated that Li et al. confirmed that rutin could facilitate autophagy by the inhibition of the PI3K/Akt axis, which diminishes macrophage inflammation and the formation of foam cells, leading to the decrease in inflammatory factors expression, thereby slowing down the formation of AS (68). Ginsenoside Rb1 was demonstrated to induce autophagy of endothelial cell and the inhibition of cell senescence by deacetylating Beclin-1 through increased SIRT1 expression, thereby preventing the progression of AS (69). In a study by Zhu's team, Salidroside (SAL) was found to reverse the expression of SIRT1 and FOXO1, which were lessened by ox-LDL, and enhance autophagy, thereby protecting endothelial cells exposed to ox-LDL (70). 13-Methylberberine (13-MB) is a novel compound of TCM extracted from berberine (BBR). The promotion of autophagy by 13-MB involves an enhancement in LC3-II and a reduction in SQSTM1/p62. At the same time, in vascular endothelial cells, 13-MB inhibits the activated NLRP3 inflammasome by promoting mitosis and decreasing ROS levels, thus improving dysfunctional vascular endothelial and slowing down the development of AS (71). Cao et al. confirmed that artemisinin can stimulate the AMPK/mTOR/ULK1 axis, initiate macrophage autophagy, and impede the macrophage foam cell formation process, thereby alleviating AS lesions (33). Tao et al. (72) constructed an *in vitro* foam cell model using oxidized low-density lipoprotein. This study found that gastrodin rejuvenates the lysosomal activity in macrophages and enhances autophagy by activating the AMPK/Forkhead box transcription factor O1/transcription factor EB signaling pathway. Activating autophagy can further reduce the IL-1β and IL-18 expression and the organization of foam cells, thus exerting an anti-atherosclerotic effect. Apigenin activates the Atg5/Atg7-dependent autophagy pathway in oxidized low-density lipoprotein (ox-LDL)-pretreated macrophages by modulating autophagosome formation. Notably, autophagy inhibition potentiates apigenin-induced macrophage apoptosis, consequently attenuating pro-atherogenic inflammation (73).

Gynostemma pentaphyllum (GP) can restore the autophagic flux damaged by ox-LDL, and reverse the reduction of LC3II and the accumulation of p62. GP reduces the requirement of ox-LDL and the formation of foam cells by enhancing Sirt1-FOXO1-mediated autophagic flux, indicating the potential of this compound in the therapy of AS (74).

Zhou et al. (75) revealed that Shenyuandan Capsule (SYDC) initiates macrophage autophagy, reduces their conversion into foam cells, and hinders the amassment of lipid droplets in cells via the regulation of PI3K/Akt/mTORC1 axis, thereby slowing down the formation of AS. Lai et al. (76) also found in a mouse experiment that SYDC may induce macrophage autophagy by the regulation of methylated levels in the promoter region of *Atg13* within genomic DNA, thereby slowing down AS in model mice. In Cao's experiment (77), it was found that a medium or high dose of Xinmaikang (XMK) can significantly reduce lipids, pro-inflammatory cytokines, plaques, and ROS levels to promote mitophagy. XMK inhibits AS by up-regulating PINK1/Parkin-mediated macrophage mitophagy. Zhang et al. (78) found that Zhishi - Xiebai-Guizhi Decoction (ZSXBGZD) may regulate the AGE-RAGE pathway. Exerting a palliative and therapeutic effect on AS through the down-regulation of AGE and HMGB1, with the release of HMGB1 leading to the up-regulation of autophagy and the down-regulation of apoptosis. It has also been found that Yi Mai Granule (YMG) may alleviate AS by the activation of mitochondrial autophagy through the downregulation of miRNA-125a-5p, upregulation of the Pink1-Mfn2-Parkin pathway, and regulation of vasoconstrictor cytokines, pro-inflammatory factors, and lipids (79). The Huayu-Qutan Formula (HYQT) affects the mTOR/TFEB axis, helps the entry of TFEB into the nucleus, and also increases the downstream ABCA1 and SCARB1 protein levels of mTOR/TFEB. Therefore, HYQT may depend on the mTOR/TFEB/ABCA1-SCARB1 signaling axis to promote macrophage autophagy and impede the organization of foam cell, thereby achieving the effect of treating AS (80). Huanglian-Jiedu-Tang (HLJDD) increases autophagy of the PI3K/Akt axis to suppress the formation of AS *in vivo* and foam cell *in vitro* (81) (Table 1).

**Table 1:** Traditional Chinese medicine targets autophagy to combat atherosclerosis.

Disease	Modes of autophagy	Name	Targets and mechanisms	References
Chinese medicine compound				
Atherosclerosis	Induce autophagy	Icariin	Enhance the intracellular TFEB/Lamin B1 and LC3II/I expression under exposure to ox-LDL, which enhancing autophagy, and inhibiting ferroptosis.	(67)
		Rutin	Facilitate autophagy by the inhibition of PI3K/Akt axis, which diminishes macrophage inflammation and the formation of foam cell.	(68)
		Ginsenoside Rb1	Induce autophagy of endothelial cell and the inhibition of cell senescence by deacetylating Beclin-1 through increased SIRT1 expression.	(69)
		Salidroside (SAL)	Reverse the expression of SIRT1 and FOXO1, which were lessened.	(70)
		13-Methylberberberine (13-MB)	Up—regulate the accumulation of LC3 - II and reduce the level of SQSTM1/p62 to promote autophagy, induce mitosis and reduce ROS production to inhibit the activation of the NLRP3 inflammasome in vascular endothelial cells.	(71)
		Artemisinin	Activate the AMPK/mTOR/ULK1 pathway, initiate macrophage autophagy, and impede the macrophage foam cell formation process.	(33)
		Asparagine	Activate the AMPK/Forkhead box transcription factor O1/transcription factor EB signaling pathway. Activating autophagy can further reduce the IL-1β and IL-18 expression and the organization of foam cells.	(72)
		Apigenin	Activate the Atg5/Atg7-dependent autophagy pathway in oxidized low-density lipoprotein (oxLDL)-pretreated macrophages by modulating autophagosome formation.	(73)
Chinese herbal extract				
Atherosclerosis	Induce autophagy	Gynostemma pentaphyllum (GP)	Restore the autophagic flux damaged by ox-LDL, and reverse the reduction of LC3II and the accumulation of p62. meanwhile reduces the requirement of ox-LDL and the formation of foam cell by enhancing Sirt1-FOXO1-mediated autophagic flux, indicating the potential of this compound in the therapy of atherosclerosis.	(74)
Chinese Patent Medicine and Chinese Herbal Formul				
Atherosclerosis	Induce autophagy	Shenyuandan Capsule (SYDC)	Activate PI3K/Akt/mTORC1 pathway pathway, promoter region Atg13, initiates macrophage autophagy and reduces foam cell formation.	(75, 76)
		Xinmaikang (XMK)	Upregulate the PINK1/Parkin pathway reduces plaque, lipids, pro-inflammatory cytokines and ROS	(77)
		Zhishi - Xiebai - Guizhi Decoction (ZSXBGZD)	Modulate the AGE-RAGE signaling pathway and enhance cellular autophagy.	(78)
		Yi Mai Granule (YMG)	Down-regulate miRNA-125a-5p activates mitochondrial autophagy and up-regulation of the Pink1-Mfn2-Parkin pathway regulates pro-inflammatory factors, vasoconstrictor cytokines, and lipids.	(79)
		Huayu - Qutan Formula (HYQT)	Regulates mTOR/TFEB signaling pathway and promotes cellular autophagy.	(80)
		Huanglian - Jiedu - Tang (HLJDD)	Induce autophagy of the PI3K/Akt axis to suppress the formation of AS *in vivo* and foam cells *in vitro*.	(81)

#### 4.1.2 Diabetic cardiomyopathy

Su et al. (82) revealed that Scutellarin (SCU) may relieve DCM by the regulation of autophagy-related factors with an enhancement of Beclin-1, LC3-I, and LC3-II and a decrease of caspases, Bax, and Cyt-C in cardiomyocytes. *in vivo* studies revealed that Cinnamaldehyde (CA) ameliorated diabetic cardiomyopathy (DCM) in rats, as evidenced by improved BNP levels and electrocardiographic profiles. Mechanistically, CA suppressed AMPK-α2, PGC-1*α*, and PPAR*α* expression while activating autophagy via mTOR pathway stimulation (increased p-mTOR/mTOR and LC3II/LC3I ratios) (83).

Epigallocatechin-3-gallate (EGCG) is a bioactive polyphenol extracted from tea with anti-oxidant, anti-inflammatory, and anti-apoptotic properties. It was revealed to reduce excessive extracellular matrix deposition and attenuate myocardial injury and myocardial fibrosis, possibly through stimulating the AMPK/mTOR pathway, improving LC3 and Beclin-1expression, and activating autophagy, which in turn restrains the TGF-β/matrix metalloproteinase pathway (84). Rutaecarpine can activate autophagy through the transient receptor potential vanilloid receptor 1, diminish the expression of apoptotic protein caspase-3 and Bax/Bcl-2, suppress apoptosis induced by hyperglycemic levels, and relieve the symptoms of diabetic cardiomyopathy (85). Zhou et al. (86). demonstrated that tea polyphenols enhance autophagy by activating AMPK through the Ca^2+^/CaMKK/AMPK signaling pathway in both normal and obese mouse models. Ginkgo biloba extract (GBE) alleviates metabolic disorders in diabetic rats, improves the function of the heart, and reduces myocardial pathological alterations. In addition, in the hearts of diabetic rats, it was observed that GBE can reverse the decrease in the transformation of LC3B-I to LC3B-II and the excessive amassment of p62, reduced AMPK phosphorylation and enhanced in mTOR phosphorylation through the AMPK/mTOR pathway, thereby activating AMPK and suppressing mTOR to reserve autophagy (87).

One study verified that spleen-carrying, blood-activating, and knot-dissolving formula for DCM promotes energy metabolism by regulating targets, which contributes to autophagy genesis. The yunpi-huoxue-sanjie formula (YP-SJ) formula increased FoxO1, Atg7, Beclin 1, and LC3 expressions, and decreased phosphorylated FoxO1 expression *in vitro* and *in vivo* (88). Rice wine polyphenols (RWPH) and polypeptides (RWPE) are the active compounds derived from Chinese rice wine. Another research revealed that in a mouse experiment treated by RWPH and RWPE, an increase of the LC3BII/LC3BI ratio and ATG5 expression and a reduced SQSTM1/p62 expression were observed through promoting autophagy, which improves the cardiac function of DCM mice (89). In the cellular experiments conducted by Wang et al. (90), the combination of ginsenosides (GN) and metformin (MET) demonstrated enhanced suppression of reactive oxygen species (ROS) overproduction while modulating PINK1/Parkin-mediated mitophagy, ultimately improving cardiomyocyte function and ameliorating diabetic cardiomyopathy (Table 2).

**Table 2:** Traditional Chinese medicine targets autophagy to combat diabetic cardiomyopathy.

Disease	Modes of autophagy	Name	Targets and mechanisms	References
Chinese medicine compound				
Diabetic cardiomyopathy	Induce autophagy	Scutellarin (SCU)	By the regulation of autophagy-related factors with an enhancement of Beclin-1, LC3-I and LC3-II and a decrease of caspases, Bax and Cyt-C in cardiomyocytes.	(82)
		Cinnamaldehyde (CA)	Activate autophagy via mTOR pathway stimulation (increased p-mTOR/mTOR and LC3II/LC3I ratios).	(83)
	Inhibit autophagy	Asiaticoside (ASI)	Activate the AMPK/Nrf2 pathway to improve Atg5, HO-1, Beclin1, p-AMPK and nuclear Nrf2 expressions, inhibit autophagy and oxidative stress.	(91)
Chinese herbal extract				
Diabetic cardiomyopathy	Induce autophagy	Epigallocatechin-3-gallate (EGCG)	Stimulate the AMPK/mTOR pathway, improving LC3 and Beclin-1expression, and activating autophagy.	(84)
		Rutaecarpine	Activate autophagy through the transient receptor potential vanilloid receptor 1, diminish the expression of apoptotic protein caspase-3 and Bax/Bcl-2.	(85)
		Tea polyphenols	enhance autophagy by activating AMPK through the Ca^2+^/CaMKK/AMPK signaling pathway.	(86)
		Ginkgo biloba extract (GBE)	Reverse the decrease in the transformation of LC3B-I to LC3B-II and the excessive amassment of p62, reduced AMPK phosphorylation and enhanced in mTOR phosphorylation through the AMPK/mTOR pathway, thereby activating AMPK and suppressing mTOR to reserve autophagy.	(87)
	Inhibit autophagy	Tea polyphenols	Inhibit autophagy levels by being able to down-regulate LC3-II/Ⅰ and Beclin-1 protein expression levels.	(86)
Chinese Patent Medicine and Chinese Herbal Formul				
Diabetic cardiomyopathy	Induce autophagy	Yunpi-Huoxue-Sanjie formula (YP-SJ)	Up-regulate FoxO1, Atg7, Beclin 1, and LC3, and decreases the expression of phosphorylated.FoxO1.	(88)
		RWPH and RWPE	Increase LC3BII/LC3BI ratio and ATG5 expression, and decrease SQSTM1/p62 expression, promoting autophagy.	(89)
		Ginsenosides (GN)	Upregulate PINK1/Parkin-mediated autophagy suppresses ROS generation.	(90)
	Inhibit autophagy	Shengjie Tongyu Decoction (SJTYD)	Suppress autophagy via activation of the lncRNA H19, ROS, and the PI3K/Akt/mTOR signaling pathway.	(89)

#### 4.1.3 Myocardial ischemia-reperfusion injury

Hu et al. (92) confirmed that Ginsenoside Rb1 can boost the expression of ubiquitin-activating enzyme E3, FUNDC1, and PINK1 in the mitochondria of H9C2 cardiomyocytes of acute MI model mice, inhibit the p62 expression, improve the ratio of LC3-II/LC3-I and phosphorylated AMPK*α* (p-AMPK*α*)/AMPK*α* in cardiomyocytes, effectively promote mitophagy, and thus improve the cardiomyocyte damage of model mice. This suggests that the AMPK*α* pathway may have involvement in the protective effect of Ginsenoside Rb1 on acute MI injury. Isoliquiritigenin (ISO) is one of the bioactive components isolated from the roots of Glycyrrhiza plants. ISO induces autophagy by regulating the AMPK/mTOR/ULK1 signal, up-regulates the levels of Beclin1, LC3II/LC3I and p-AMPK/AMPK, and inhibits the protein expression of P62, p-mTOR/mTOR and p-ULK1 (S757)/ULK1, thereby promoting myocardial IR injury as a candidate drug for the therapy (93).

Xu et al. (32) indicated that Cordycepin promotes autophagy during Hypoxia/Reoxygenation (H/R) injury in mice by up-regulating the AMPK/mTOR pathway, thereby decreasing myocardial I/R injury. It was found that crocin significantly promoted autophagy during MI with AMPK activation (94).

Experiments have found that Shexiang Baoxin Pill (SBP) can ameliorate myocardial I/R injury and lessen the infarct area in mice. SBP boosts autophagic flux and the production of autophagosome via the regulation of the interactive effect of mmu_circ_0005874/mmu-miR-543-3p/Map3k8, thus facilitating autophagosome degradation, suppressing pyroptosis and oxidative stress, and improving the survival of cardiomyocytes after I/R injury (95). Si-Miao-Yong-An Decoction (SMYAD) promoted LC3B-II/LC3B-I expression and lessened p-mTOR/mTOR expression in cardiomyocytes of H/R mice, and reduced caspase 1, NLRP3, and IL-1βexpression. Activating autophagy and inhibiting cellular charring exerted a protective effect against ischemia/reperfusion injury in cardiomyocytes, thereby improving cardiac function (96) (Table 3).

**Table 3:** Traditional Chinese medicine targets autophagy to combat myocardial ischemia-reperfusion injury.

Disease	Modes of autophagy	Name	Targets and mechanisms	References
Chinese medicine compound				
Myocardial ischemia-reperfusion injury	Induce autophagy	Ginsenoside Rb1	Boost the expression of ubiquitin-activating enzyme E3, FUNDC1, and PINK1 in the mitochondria of H9C2 cardiomyocytes of acute myocardial ischemia model mice, inhibit the p62 expression, improve the ratio of LC3-II/LC3-I and phosphorylated AMPK*α* (p-AMPKα)/AMPKα in cardiomyocytes.	(92)
		Isoliquiritigenin (ISO)	Induce autophagy by regulating the AMPK/mTOR/ULK1 signal, up-regulates the levels of Beclin1, LC3II/LC3I and p-AMPK/AMPK, and inhibits the protein expression of P62, p-mTOR/mTOR and p-ULK1 (S757)/ULK1.	(93)
	Inhibit autophagy	Danshensu-Tetramethylpyrazine Conjugate DT-010	Inhibit autophagy, scavenges ROS and attenuates oxidative stress via the AMPK/mTOR/Ulk1 axis.	(97)
		Danpihenol	Down-regulate Beclin1, p62, and LC3-I/LC3-II expressions in the myocardium via PI3K/Akt/mTOR signaling pathways, and significantly inhibit myocardial I/R-induced autophagy.	(98)
Chinese herbal extract				
Myocardial Ischemia-Reperfusion Injury	Induce autophagy	Cordycepin	Promote autophagy during H/R injury by upregulating the AMPK/mTOR pathway.	(32)
		Crocin	During myocardial ischemia: activation of AMPK promotes autophagy.	(94)
	Inhibit autophagy	Resveratrol (RES)	Inhibit autophagy through downregulation of P62 and upregulation of LC3-II/LC3-I through the DJ-1/MEKK1/JNK axis.	(99)
		Crocin	During reperfusion: activation of Akt through AMPK/m TOR and Akt/mTOR signaling pathways inhibits autophagy.	(94)
		Galangin (Gal)	Decrease LC3-II and p62 levels to improve autophagy by the PI3K/Akt/mTOR axis.	(100)
Chinese patent medicine and Chinese herbal formul				
Myocardial Ischemia-Reperfusion Injury	Induce autophagy	Shexiang Baoxin Pill (SBP)	Boost autophagic flux and the production of autophagosome via the regulation of the interactive effect of mmu_circ_0005874/mmu-miR-543-3p/Map3k8, thus facilitating autophagosome degradation.	(95)
		Si-Miao-Yong-An Decoction (SMYAD)	Promote LC3B-II/LC3B-I expression and lessened p-mTOR/mTOR expression in cardiomyocytes of H/R mice, and reduced caspase 1, NLRP3, and IL-1βexpression.	(96)
	Inhibit autophagy	Rhodiola Injection	Regulate the AMPK/mTOR pathway inhibits autophagy, decreases p-AMPK and LC3II expression, increases p-mTOR expression, and inhibits ROS production.	(58)
		Suxiao Jiuxin Pill (SJP)	Dependent on its capability of up-regulating ALKBH5 through miR-193a-3p, which enhanced the phosphorylated mTOR and P62 expression, as well as reduced Beclin-1 expression and the LC3 (II/I) ratio.	(101)
		Tao Hong Si Wu Tang	Up-regulate LC3, IL-6, IL-18 and IL-1β expressions, and down-regulating p62 and NLRP3 expressions.	(102)

#### 4.1.4 Heart failure

Disturbed energy metabolism in cardiomyocytes is a key cause of the pathology of HF, ultimately leading to a poor prognosis (103). Asiatic Acid improves the homeostasis of energy metabolism in cardiomyocytes through the activation of mitophagy (104), promotes mitochondrial autophagy, alleviates mitochondrial homeostatic dysregulation, and can enhance LC3-II expression through ischemic myocardial mitochondrial phagolysosomes *in vivo*. It has also been found that enhancing PINK1/Parkin-mediated mitophagy by bromelain offers protection for cardiac function in patients with HF. Guan et al. (105) indicated a decrease in PINK1, Parkin, and LC3-II levels, along with an increase of p62 in an HF mouse model. However, Ginsenoside Rg1 could reverse the expression of these proteins. Furthermore, the SIRT1 gene expression increased, indicating that Ginsenoside Rg1 can mediate mitophagy by the stimulation of the SIRT1/PINK1/Parkin signaling axis, remove damaged mitochondria, and protect cardiomyocytes. The research of Wang et al. (106) found that Morinda officinalis (MO) can treat HF by modulating the FoxO3 signaling pathway to up-regulate LC3B, P62 (autophagy markers), and p-FoxO3.

In Zhang's rat experiment, it was confirmed that Tanshinone IIA can reduce phosphorylated mTOR, p62, and S6K1 via the AMPK-mTOR pathway, thus enhancing autophagy to lessen myocardial ischemic injury and protecting cardiac function (107).

In the experimental research of Lv et al. Qishen Yiqi Dropping Pills (QSYQDP) can suppress the activity of the PI3K/Akt/mTOR axis, increase the expressions of Beclin-1 and LC3II, activate myocardial autophagy, and improve aortic-constriction-induced cardiac fibrosis and HF in rats (108). In Liu's article, it was mentioned that Shenkui Tongxin Granules can modulate the AMPK-mtTFA-PINK1 signaling axis, promote cardiomyocyte mitophagy, enhance mitochondrial biogenesis, alleviate mitochondrial injury, and reduce energy-metabolism disorders, thus alleviating the advancement of HF (109). In Dong's mouse experiments, Xinyang Tablet (XYT) was found to prevent pressure overload-induced heart failure by modulating receptor-interacting protein kinase 3 (RIPK3)/FUNDC1-mediated mitochondrial unfolded protein response and mitophagy (110). In addition, the traditional Chinese medicine compound Nuanxinkang was also able to improve symptoms of HF by modulating PINK1/Parkin-mediated mitochondrial autophagy (111) (Table 4).

**Table 4:** Traditional Chinese medicine targets autophagy to combat heart failure.

Disease	Modes of autophagy	Name	Targets and mechanisms	References
Chinese medicine compound				
Heart failure	Induce autophagy	Asiatic Acid	Upregulate LC3-II expression promotes mitochondrial autophagy.	(103, 104)
		Ginsenoside Rb1	Mediate mitophagy by the stimulation of the SIRT1/PINK1/Parkin signaling axis, remove damaged mitochondria.	(105)
		Morinda officinalis (MO)	Modulate the FoxO3 signaling pathway to up-regulate LC3B, P62 (autophagy markers), and p-FoxO3.	(106)
	Inhibit autophagy	Astragaloside IV	Inhibit autophagy by down-regulating Beclin1 and LC3II/LC3I mRNA and protein expression levels and up-regulating p62 levels.	(112)
		Ophiopogon japonicus D (OP - D)	Inhibit DOX produced ROS-induced excessive autophagy in H9C2 cells and myocardium, controlled autophagic vacuole number and LC3-II/I expression, and attenuated MMP destruction.	(113)
		Panax ginseng stem and leaf saponins	Decrease LC3B, Beclin-1 and p62 expressions through the PI3K/Akt/mTOR axis, and suppressing aberrant autophagy and apoptosis.	(114)
Chinese herbal extract				
Heart failure	Induce autophagy	Tanshinone IIA	Reduce phosphorylated mTOR, p62, and S6K1 via the AMPK-mTOR pathway, thus enhancing autophagy.	(107)
Chinese Patent Medicine and Chinese Herbal Formul				
Heart failure	Induce autophagy	Qishen Yiqi Dropping Pills (QSYQDP)	Suppress the activity of the PI3K/Akt/mTOR axis, increase the expressions of Beclin-1 and LC3II, activate myocardial autophagy.	(108)
		Shenkui Tongxin Granules	Regulate AMPK-mt TFA-PINK1 signaling axis promotes mitochondrial autophagy in cardiomyocytes.	(109)
		Xin Yang Tablet (XYT)	Adjust the RIPK3/FUNDC1 pathway to regulate autophagy.	(110)
		Nuanxinkang	Regulate PINK1/Parkin pathway to regulate autophagy.	(111)
	Inhibit autophagy	Qiliqiangxin (QLQX)	Suppress the ROS/AMPK/mTOR axis to reduce LC3II and p62 levels while attenuating apoptosis and autophagic cell death.	(115)

### 4.2 Autophagy inhibition in the treatment of cardiovascular diseases by Chinese medicine compounds, herbal extracts, patent medicines, and formulations

#### 4.2.1 Diabetic cardiomyopathy

Asiaticoside (ASI) can activate the AMPK/Nrf2 pathway to improve Atg5, HO-1, Beclin1, p-AMPK, and nuclear Nrf2 expressions, inhibiting autophagy and oxidative stress, thereby relieving myocardial damage of DCM (91).

Consistent with Zhou's experiments, the DCM group exhibited excessive autophagy activation, which exacerbated cardiomyocyte apoptosis and cardiac dysfunction, suggesting the potentially detrimental role of autophagy under pathological conditions. Notably, tea polyphenol intervention effectively suppressed this maladaptive autophagic response, preventing excessive cardiomyocyte apoptosis/necrosis while concurrently modulating glucolipid metabolism (86).

In Wang's mouse experiments (89), the Shengjie Tongyu Decoction (SJTYD) group showed significantly lower expression of autophagy markers (LC3A-II, Beclin-1, and Atg7) compared to the model group, indicating that SJTYD inhibits cardiomyocyte autophagy. This protective effect against diabetic cardiomyopathy (DCM) is achieved through suppression of autophagy via activation of the lncRNA H19, ROS, and the PI3K/Akt/mTOR signaling pathway (Table 2).

#### 4.2.2 Myocardial ischemia-reperfusion injury

Danshensu-Tetramethylpyrazine Conjugate DT-010 exhibits a cardioprotective effect based on suppressing autophagy via the AMPK/mTOR/Ulk1 axis. It scavenges ROS and alleviates oxidative stress using the t-BHP-induced H9c2 cardiomyocyte-like cell model with oxidative damage, thus improving MIRI (97). Danpihenol is an active phenolic compound extracted from the root bark of peony bark (Paeonia × suffruticosa Andrews) (Paeoniaceae), serving as an analgesic, antipyretic, and anti-inflammatory agent in TCM. Danphenol can down-regulate Beclin1, p62, and LC3-I/LC3-II expressions in the myocardium via PI3K/Akt/mTOR signaling pathways, and significantly inhibit myocardial I/R-induced autophagy (98).

Resveratrol (RES) prevented cardiac ischemia-reperfusion by inhibiting autophagy through downregulation of P62 and upregulation of LC3-II/LC3-I through the DJ-1/MEKK1/JNK axis and also induced a decrease in cardiac ischemia-reperfusion-stimulated MAPK/MEKK1 and JNK phosphorylation, as well as increased cell viability (99). Similarly, crocin was found to suppress excessive autophagy and activate Akt during reperfusion, suggesting its bidirectional regulation of autophagy through both the AMPK/mTOR and Akt/mTOR signaling pathways, thereby exerting a protective effect against ischemia-reperfusion injury (93). In the experiment of Zhang et al. (100), it was known that Galangin (Gal) decreases LC3-II and p62 levels to improve autophagy by the PI3K/Akt/mTOR axis, and retains the damaged contractile function after myocardial I/R and reduces the infarct area in an autophagy-dependent manner.

In addition, research shows that Rhodiola Injection can inhibit autophagy by regulating the AMPK/mTOR pathway, reducing the expression of p-AMPK and LC3Ⅱ, increasing the expression of p-mTOR, inhibiting the production of ROS, and alleviating MIRI (58). Wang et al. (101) identified that the anti-autophagic effect of Suxiao Jiuxin Pill (SJP) was dependent on its capability of up-regulating ALKBH5 through miR-193a-3p, which enhanced the phosphorylated mTOR and P62 expression, as well as reduced Beclin-1 expression and the LC3 (II/I) ratio, thereby attenuating myocardial I/R injury. In another study, Tao Hong Si Wu Tang was found to decrease inflammatory response and cellular death in mice by up-regulating LC3, IL-6, IL-18, and IL-1β expressions, and down-regulating p62 and NLRP3 expressions, which was reversed by 3-methyladenine, an inhibitor of autophagy (102) (Table 3).

#### 4.2.3 Heart failure

In lipopolysaccharide-induced rats, Astragaloside IV can perform the downregulation of miRNA-1, and significantly reduce Beclin1 and LC3II/LC3I expressions in heart tissues, while significantly increasing P62 expression. This inhibits cell autophagy and prevents lipopolysaccharide-induced cardiac dysfunction and HF (112). Ophiopogon japonicus D (OP-D) can improve the antioxidant defense mechanisms in the cardiovascular system and cardiomyocytes. It alleviates cardiotoxicity by inhibiting the excessive autophagy resulteing from ROS produced by DOX in H9C2 cells and the myocardium, controls the quantities of autophagic vacuoles and the expression of LC3-II/I, and significantly reduces MMP damage (113). Another study found that the saponin extracted from Panax ginseng stem and leaves is a beneficial Chinese medicine that activates blood circulation and stops bleeding, and is the main bioactive ingredient in the therapy of CVDs containing cardiac arrhythmia, ischemia-reperfusion injury, and myocardial hypertrophy. Panax ginseng stem and leaf saponins ameliorate cardiac hypertrophy by down-regulating LC3B, Beclin-1 and p62 expressions through the PI3K/Akt/mTOR axis, and suppressing aberrant autophagy and apoptosis, thus exerting a cardioprotective effect in rats (114).

Fan et al. (115) revealed that Qiliqiangxin (QLQX), a compounded herbal formulation, was applied for treating congestive HF. QLQX exerts protective impact by suppressing the ROS/AMPK/mTOR axis to reduce LC3II and p62 levels while attenuating apoptosis and autophagic cell death (Table 4).

## 5 Summary and outlook

TCM is progressively gaining global recognition, with increasing acceptance worldwide. The therapeutic value characteristics of TCM in CVDs lie in its multi-component, multi-target mechanisms, coupled with low adverse effects, high safety profiles, and cost-effectiveness. Furthermore, TCM has garnered robust support from evidence-based medicine, establishing itself as a vital approach to CVDs treatment. In clinical practice, patients with AS typically receive conventional Western therapies, including antiplatelet aggregation agents, lipid-lowering drugs, and antihypertensive medications. However, long-term use of these pharmaceuticals may lead to hepatotoxicity, renal impairment, and gastrointestinal bleeding. In contrast, TCM demonstrates significant potential in reducing major adverse cardiovascular events (MACE) while mitigating drug-induced organ damage. DCM, a distinctive myocardial pathology specific to diabetes mellitus, requires comprehensive management strategies that extend beyond conventional cardiovascular therapies. The cornerstone of DCM treatment involves rigorous glycemic control, with insulin and other hypoglycemic agents mandating strict clinical supervision by physicians to prevent complications such as hypoglycemia. TCM exhibits multi-target therapeutic effects, where single herbal extracts, proprietary Chinese medicines, or compound formulations can simultaneously address hyperglycemia and ameliorate myocardial pathology. While TCM cannot entirely supplant conventional antidiabetic medications or insulin therapy, it serves as an effective adjuvant treatment that synergizes with mainstream therapeutic approaches. MIRI in TCM theory is attributed to the pathological patterns of qi deficiency, blood stasis, and phlegm-dampness accumulation. The TCM therapeutic principles of replenishing qi, nourishing blood, activating blood circulation, and resolving stasis are particularly beneficial for cardiac functional recovery post-reperfusion. Guided by the holistic treatment philosophy, TCM provides comprehensive therapeutic interventions targeting multiple pathological aspects simultaneously. In the management of heart failure, TCM demonstrates remarkable advantages in both safety and tolerability profiles. While conventional Western medical therapies carry risks of electrolyte disturbances, renal function deterioration, and hypotension, these adverse events occur in less than 5% of cases receiving TCM interventions.

Through research and collation, it confirmed that autophagy functions in the emergence and development of CVDs containing atherosclerosis, diabetic cardiomyopathy, MI, MIRI, HF, and hypertension. Excessive regulation of the autophagy process may induce damage to the myocardium and blood vessels. Therefore, maintaining the balance of autophagy activities is crucial for coordinating physiological activities and body health.

Despite some advancements in research in the use of TCM to intervene in autophagy for the therapy of CVDs, there are still some obvious deficiencies. Firstly, the current research on the application of TCM to modulate autophagy for the therapy of CVDs mainly focuses on cell and animal models, and lack of data in clinical practice. Secondly, although research has shown that TCM has a therapeutic efficacy on certain diseases, its specific metabolic pathways and targets have not been clearly explained. Next, the effect of autophagy changes the stage of disease development. Finding a way to balance autophagy to ensure that traditional Chinese medicine can achieve the optimal therapeutic effect by adjusting autophagy is both a challenge and the key. The investigation of TCM-mediated autophagy regulation represents a paradigm shift from traditional experience-based practice to modern precision medicine. Notwithstanding the existing challenges in standardization and mechanistic interpretation, TCM's distinctive strengths in systemic modulation and favorable safety profile provide innovative therapeutic avenues for cardiovascular disease management. Finally, the interaction between single drugs in Chinese patent medicines and TCM compound prescriptions is not very clear. In addition, autophagy may involve more signaling pathways. We have to deeply investigate the mechanism of autophagy in the advancement of CVDs to find more treatment targets for CVDs and provide a certain theoretical perspective and treatment strategies for the clinical therapeutics of CVDs. It is believed that the mechanism of TCM in regulating autophagy will be further clarified in more cardiovascular disease research.
